# Supplementary material for: Molecular Pathogenesis of Gene Regulation by the miR-150 Duplex: miR-150-3p Regulates TNS4 in Lung Adenocarcinoma
Source: Cancers (Basel). 2019 Apr 30;11(5):601. doi: 10.3390/cancers11050601 (PMC6562801; doi:10.3390/cancers11050601)
Supplement: Supplementary file 1 [file cancers-11-00601-s001.pdf]

Supplementary Materials: Molecular pathogenesis of gene regulation by the *miR-150* duplex: *miR-150-3p* regulates *TNS4* in lung adenocarcinoma

Shunsuke Misono, Naohiko Seki, Keiko Mizuno, Yasutaka Yamada, Akifumi Uchida, Hiroki Sanada, Shogo Moriya, Naoko Kikkawa, Tomohiro Kumamoto, Takayuki Suetsugu and Hiromasa Inoue

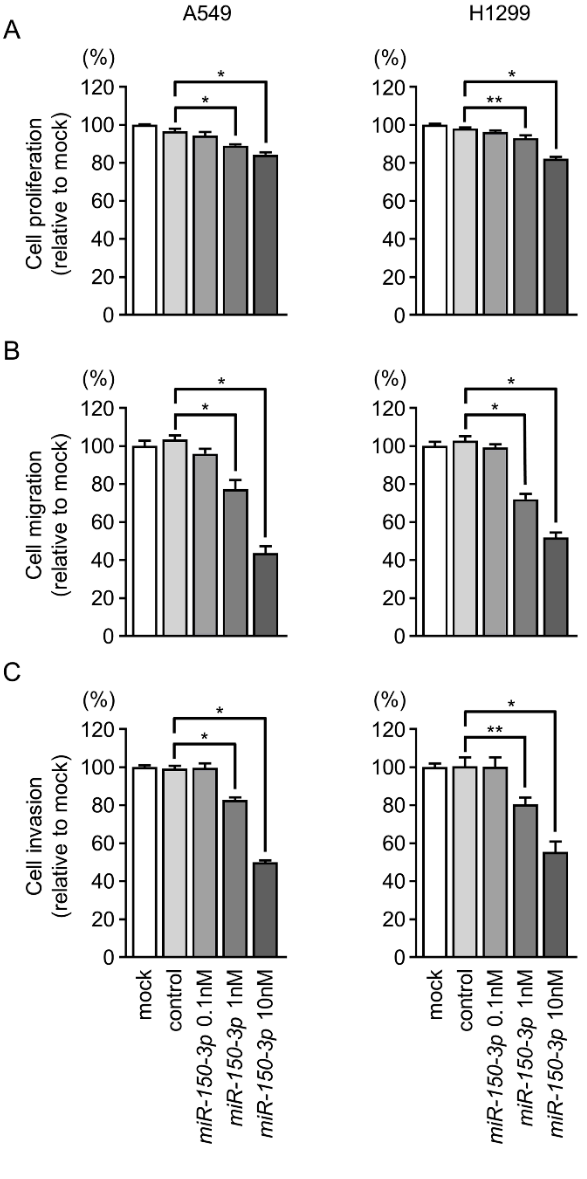

Figure S1. Functional assays of *miR-150-3p* (0.1 nM, 1 nM, 10 nM) in LUAD cells (A549 and H1299). (A-C) Cell proliferation, migration, and invasive activities were significantly blocked by ectopic expression of *miR-150-3p* with 1 nM and 10 nM concentration. * $p < 0.01$, ** $p < 0.05$.

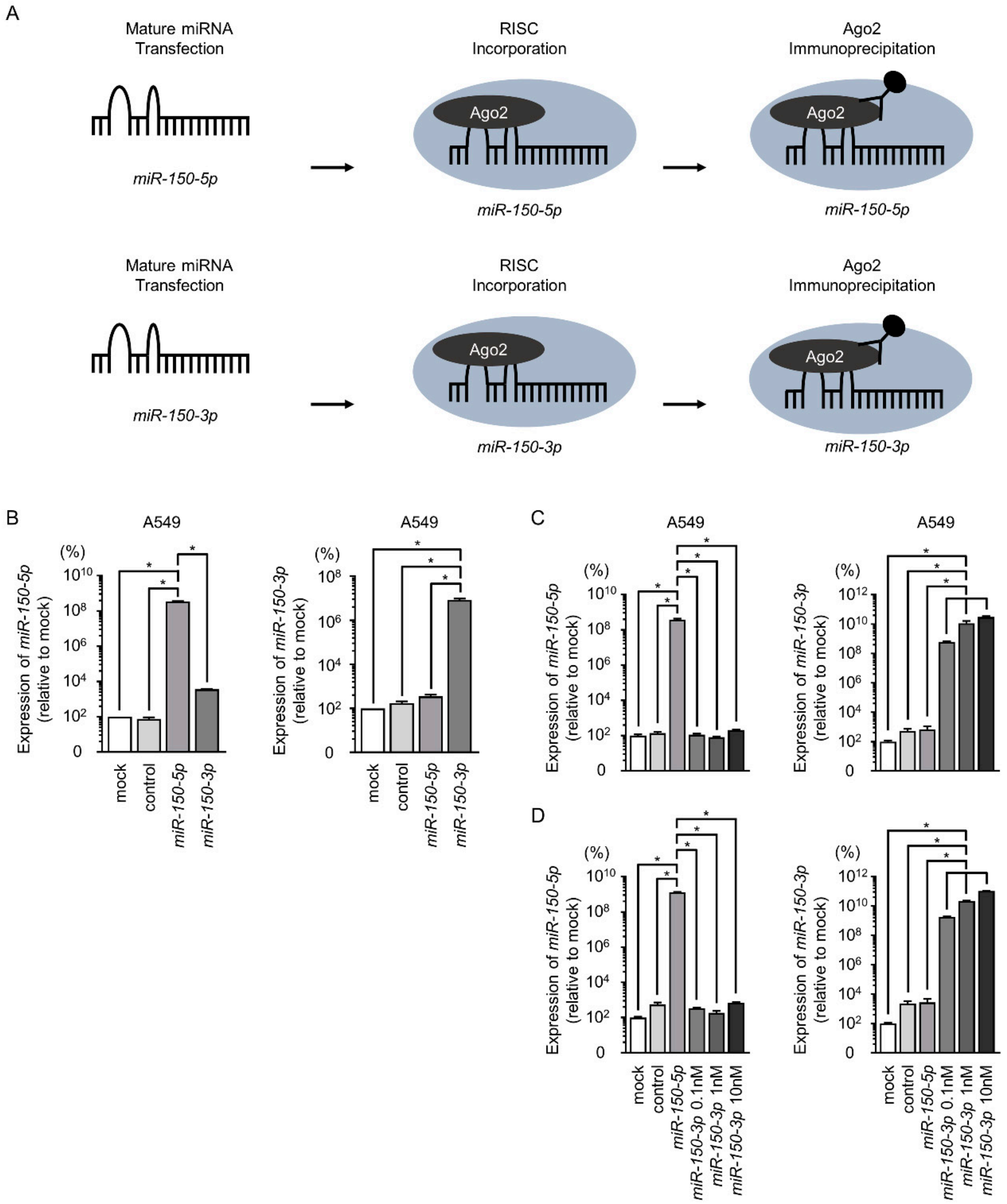

Figure S2. Both strands of *miR-150-5p* and *miR-150-3p* were incorporated into the RISC. (**A**) Schematic diagram for isolation of the Ago2-miRNA complex from the RISC. (**B**–**D**) Expression levels of *miR-150-5p* or *miR-150-3p* bound to Ago2 were measured by TaqMan RT-qPCR and normalized to the expression of *miR-26a* (**B**), *miR-16-5p* (**C**) and *miR-21-5p* (**D**), which had no effect upon *miR-150-5p* and *miR-150-3p*. * $p < 0.01$.

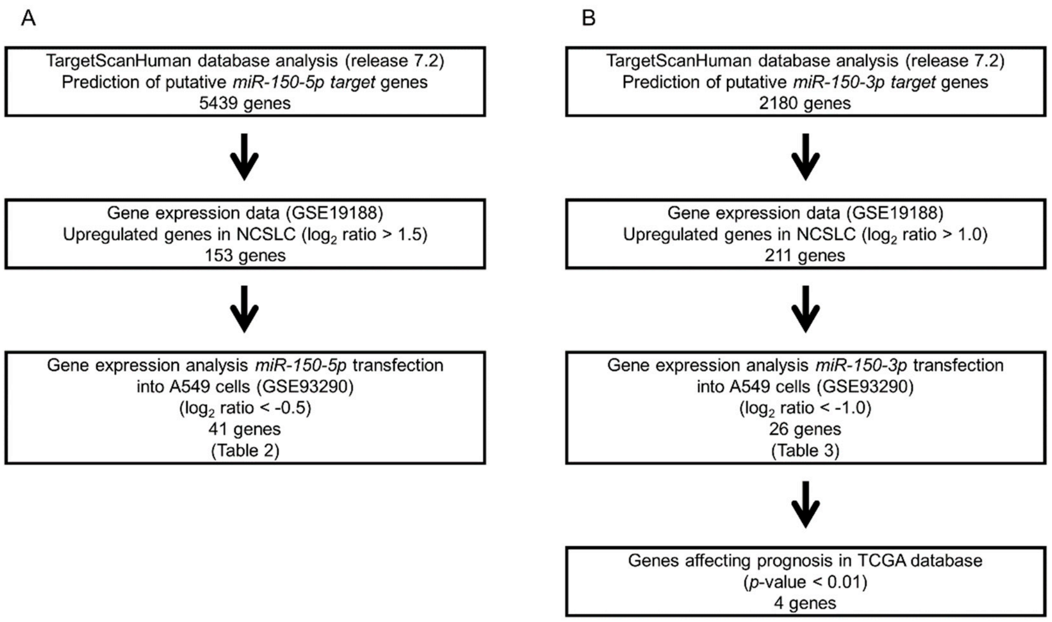

Figure S3. The strategy for identification of genes regulated by *miR-150-5p* and *miR-150-3p*. We obtained putative genes regulated by *miR-150-5p* and *miR-150-3p* using the TargetScanHuman Release 7.2 database and selected upregulated genes in non-small cell lung cancer clinical expression profiles from the GEO database (GSE19188). Then, we combined the gene expression analyses using A549 cells transfected with *miR-150-5p* or *miR-150-3p* and inspected the downregulated genes (GSE93290).

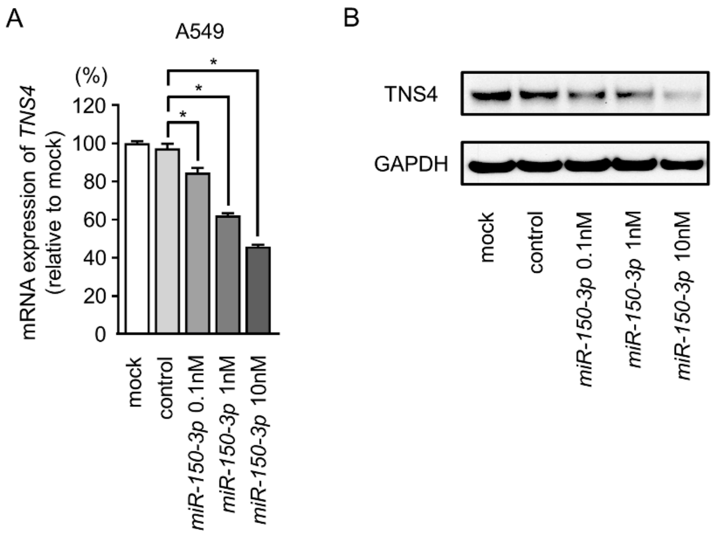

Figure S4. *TNS4* was directly suppressed at the diluted concentration of *miR-150-3p* precursor (1 nM and 0.1 nM). (**A**,**B**) *TNS4* mRNA and protein expression was reduced by *miR-150-3p* ectopic expression (48h after transfection). *GUSB* was used as an expression control. GAPDH was used as a loading control. * $p < 0.01$.

TNS4 3′UTR variant 1

GACAGAGCCTCAGGAGAACGTATGCCACCTCTTTGCGGAGTATGACATGGTCCAGCCAGC

CTCGCAGGTCATCGGCCTGGTGACTGCTCTGCTGCAGGACGCAGAAAGGATG**TAG**GGGAG

AAACTGCCTGTGCACCTAACCAACACCTCCAGGGGCCCGCTAAGGAGCCCCCCTCCACCC

CCTGAATGGGTGTGGCTTGTGGCCATATTGACAGACCAATCTATGGGACTAGGGGGATTG
GCATCAAGTTGAAACCCCATCTCTACAAAGAATACAAAAATTAGCCGGGCAAGGTAGCGC
ACCTGTGGTCCCAGGTACTCGGGAGACTGAGGCATGAGAATCCCTTGAAACTGGGAGGCG
GAAGTTGCAGTGAGCTGTGATCGTGCCACTGCACTCCAGCCTGGTTGACAGAGCAACATG
CTATCTCTAAAACAAACAAACAAACAAAAACTCAGGTTCCCACACCCTCTAAACCCTGCC
TCCTCTCAGGCTACAGAGACCTCTCCAGGAGGCTGAAGTGCCCTTACCCCGACCATCTGA
CCAGCCACCGCCCCATGCCCGTGCCCCACCGAGGGCGGAGGCTGCTCACTGCTCTGTTTT
ATCTCTGGCCTCTGATCCTGCATTCTTGTGCCAGGGCTTAGACCCAGGGCAAGGTCTTAG
ACCCAGGGCAGGGGTAGGGTTAAAGGCTTTCAACCCAGGGGCCAGTGCCTTAATTCATGC
AACAAATGTTTTCTGGCTGTGTGCTTTATTCATGTGAACCAGGAAAACAGAAAAATATGA
CAGTGTTTCCAACAGAGGTTTATAAGTGCTATGCAGGGCTGGTGGAAAGAGTAGAAACTG
AGAAAGATAAACTTTACCCACTTGAAGGGAAGGAGGGCAATGTTACCAAGAAGGTAACAT
TTGAGTTGGGTCTTCAAGGATGAATAGGAGTTCGGCATGCAAAGAGAGTTAGAAACCAAC
TTTTAGGAGTGGGGAGGGGCTCTCATGTGCTACATACAATCTGAGGCACATTATATATGC
CTAATCCCATTTTACAGATTAGAAAACTGGGGCTCAGAGGGTTAACTTGCCCACATTCAC
CTAACTGTAAATGGCAGAGAAACAGGATTTCAAGTCCATGCCCATCCTATTGCCCCAGCA
TTCACAGAAAGCAGATGGAGACATTCGTGTGTGAAGCACACAGGTATGAAAAGATGTACC
AAGTTTGGTGTGGCTCAAGTACATGGTACCTAAGGGAGTAGGTGAGAGAGAAAGCAGAAG

TNS4 3'UTR variant 2

GACAGAGCCTCAGGAGAACGTATGCCACCTCTTTGCGGAGTATGACATGGTCCAGCCAGC
CTCGCAGGTCATCGGCCTGGTGACTGCTCTGCTGCAGGACGCAGAAAGGATG**TAG**GGGAG
AGACTGCCTGTGCACCTAACCAACACCTCCAGGGGCTCGCTAAGGAGCCCCCCTCCACCC
CCTGAATGGGTGTGGCTTGTGGCCATATTGACAGACCAATCTATGGGACTAGGGGGATTG
GCATCAAGTTGACACCCTTGAACCTGCTATGGCCTTCAGCAGTCACCATCATCCAGACCC
CCCGGGCCTCAGTTTCCTCAATCATAGAAGAAGACCAATAGACAAGATCAGCTGTTCTTA
GATGCTGGTGGGCATTTGAACATGCTCCTCCATGATTCTGAAGCATGCACACCTCTGAAG
ACCCCTGCATGAAAATAACCTCCAAGGACCCTCTGACCCCATCGACCTGGGCCCTGCCCA
CACAACAGTCTGAGCAAGAGACCTGCAGCCCCTGTTTCGTGGCAGACAGCAGGTGCCTGG
CGGTGACCCACGGGGCTCCTGGCTTGCAGCTGGTGATGGTCAAGAACTGACTACAAAACA
GGAATGGATAGACTCTATTTCCTTCCATATCTGTTCCTCTGTTCCTTTTCCCACTTTCTG
GGTGGCTTTTTGGGTCCACCCAGCCAGGATGCTGCAGGCCAAGCTGGGTGTGGTATTTAG
GGCAGCTCAGCAGGGGGAACTTGTCCCCATGGTCAGAGGAGACCCAGCTGTCCTGCACCC
CCTTGCAGATGAGTATCACCCCATCTTTTCTTTCCACTTGTTTTTTATTTTTATTTTTTT
TGAGACAGAGTCTCACTGTCACCCAGGCTGAACTGCAGTGGTGTGATCTAGGCTCACTGC
AACCTCCACCTCCCAGGTACTCAGGAGACTGAGGCATGAGAATCCCTTGAAACTGGGAGG
CGGAAGTTGCAGTGAGCTGTGATCGTGCCACTGCACTCCAGCCTGGTTGACAGAGCAACA
TGCTATCTCTAAAACAAACAAACAAACAAAAACTCAGGTTCCCACACCCTCTAAACCCTG
CCTCCTCTCAGGCTACAGAGACCTCTCCAGGAGGCTGAAGTGCCCTTACCCCGACCATCT
GACCAGCCACCGCCCCATGCCCGTGCCCCACCGAGGGCGGAGGCTGCTCACTGCTCTGTT
TTATCTCTGGCCTCTGATCCTGCATTCTTGTGCCAGGGCTTAGACCCAGGGCAAGGTCTT
AGACCCAGGGCAGGGGTAGGGTTAAAGGCTTTCAACCCAGGGGCCAGTGCCTTAATTCAT
GCAACAAATGTTTTCTGGCTGTGTGCTTTATTCATGTGAACCAGGAAAACAGAAAAATAT

GACAGTGTTTCCAACAGAGGTTTATAAGTGCTATGCAGGGCTGGTGGAAAGAGTAGAAAC
TGAGAAAGATAAACTTTACCCACTTGAAGGGAAGGAGGGCAATGTTACCAAGAAGGTAAC
ATTTGAGTTGGGTCTTCAAGGATGAATAGGAGTTCGGCATGCAAAGAGAGTTAGAAACCA
ACTTTTAGGAGTGGGGAGGGGCTCTCATGTGCTACATACAATCTGAGGCACATTATATAT
GCCTAATCCCATTTTACAGATTAGAAAACTGGGGCTCAGAGGGTTAACTTGCCCACATTC
ACCTAACTGTAAATGGCAGAGAAACAGGATTTCAAGTCCATGCCCATCCTATTGCCCCAG
CATTCACAGAAAGCAGATGGAGACATTCGTGTGTGAAGCACACAGGTATGAAAAGATGTA
CCAAGTTTGGTGTGGCTCAAGTACATGGTACCTAAGGGAGTAGGTGAGAGAGAAAGCAGA

TNS4 3'UTR variant 3

GACAGAGCCTCAGGAGAACGTATGCCACCTCTTTGCGGAGTATGACATGGTCCAGCCAGC
CTCGCAGGTCATCGGCCTGGTGACTGCTCTGCTGCAGGACGCAGAAAGGATG**TAG**GGGAG
AGACTGCCTGTGCACCTAACCAACACCTCCAGGGGCTCGCTAAGGAGCCCCCCTCCACCC
CCTGAATGGGTGTGGCTTGTGGCCATATTGACAGACCAATCTATGGGACTAGGGGGATTG
GCATCAAGTTGACACCCTTGAACCTGCTATGGCCTTCAGCAGTCACCATCATCCAGACCC
CCCGGGCCTCAGTTTCCTCAATCATAGAAGAAGACCAATAGACAAGATCAGCTGTTCTTA
GATGCTGGTGGGCATTTGAACATGCTCCTCCATGATTCTGAAGCATGCACACCTCTGAAG
ACCCCTGCATGAAAATAACCTCCAAGGACCCTCTGACCCCATCGACCTGGGCCCTGCCCA
CACAACAGTCTGAGCAAGAGACCTGCAGCCCCTGTTTCGTGGCAGACAGCAGGTGCCTGG
CGGTGACCCACGGGGCTCCTGGCTTGCAGCTGGTGATGGTCAAGAACTGACTACAAAACA
GGAATGGATAGACTCTATTTCCTTCCATATCTGTTCCTCTGTTCCTTTTCCCACTTTCTG
GGTGGCTTTTTGGGTCCACCCAGCCAGGATGCTGCAGGCCAAGCTGGGTGTGGTATTTAG
GGCAGCTCAGCAGGGGGAACTTGTCCCCATGGTCAGAGGAGACCCAGCTGTCCTGCACCC
CCTTGCAGATGAGTATCACCCCATCTTTTCTTTCCACTTGTTTTTTATTTTTATTTTTTT
TGAGACAGAGTCTCACTGTCACCCAGGCTGAACTGCAGTGGTGTGATCTAGGCTCACTGC
AACCTCCACCTCCCAGGTTCAAGCAATTATCCTGCCTCAGGCTCCCAAGTAGCTGGGATT
ACAGGCATGTGCAACTCACCCAGCTAATTTTGTATTTTTAGTAGAAACATGGTGAAACCC
CATCTCTACAAAAAATACAAAAATTAGCCGGGCAAGGTAGCGCACCTGTGGTCCCAGGTA
CTCAGGAGACTGAGGCATGAGAATCCCTTGAAACTGGGAGGCGGAAGTTGCAGTGAGCTG
TGATCGTGCCACTGCACTCCAGCCTGGTTGACAGAGCAACATGCTATCTCTAAAACAAAC
AAACAAACAAAAACTCAGGTTCCCACACCCTCTAAACCCTGCCTCCTCTCAGGCTACAGA
GACCTCTCCAGGAGGCTGAAGTGCCCTTACCCCGACCATCTGACCAGCCACCGCCCCATG
CCCGTGCCCCACCGAGGGCGGAGGCTGCTCACTGCTCTGTTTTATCTCTGGCCTCTGATC
CTGCATTCTTGTGCCAGGGCTTAGACCCAGGGCAAGGTCTTAGACCCAGGGCAGGGGTAG
GGTTAAAGGCTTTCAACCCAGGGGCCAGTGCCTTAATTCATGCAACAAATGTTTTCTGGC
TGTGTGCTTTATTCATGTGAACCAGGAAAACAGAAAAATATGACAGTGTTTCCAACAGAG
GTTTATAAGTGCTATGCAGGGCTGGTGGAAAGAGTAGAAACTGAGAAAGATAAACTTTAC
CCACTTGAAGGGAAGGAGGGCAATGTTACCAAGAAGGTAACATTTGAGTTGGGTCTTCAA
GGATGAATAGGAGTTCGGCATGCAAAGAGAGTTAGAAACCAACTTTTAGGAGTGGGGAGG
GGCTCTCATGTGCTACATACAATCTGAGGCACATTATATATGCCTAATCCCATTTTACAG
ATTAGAAAACTGGGGCTCAGAGGGTTAACTTGCCCACATTCACCTAACTGTAAATGGCAG
AGAAACAGGATTTCAAGTCCATGCCCATCCTATTGCCCCAGCATTCACAGAAAGCAGATG
GAGACATTCGTGTGTGAAGCACACAGGTATGAAAAGATGTACCAAGTTTGGTGTGGCTCA

AGTACATGGTACCTAAGGGAGTAGGTGAGAGAGAAAGCAGAAGAAAAAGACAGCAACAGG

Figure S5. The nucleotide sequences of 3′UTR of *TNS4* in A549 cells. Several variants of the 3′UTR of *TNS4* existed in A549 cells. The putative binding site of *miR-150-3p* was found in 3′UTR of *TNS4*, respectively (highlighted in blue). Red font was stop codon. There was no binding site of *miR-150-5p* in 3′UTR of *TNS4*.

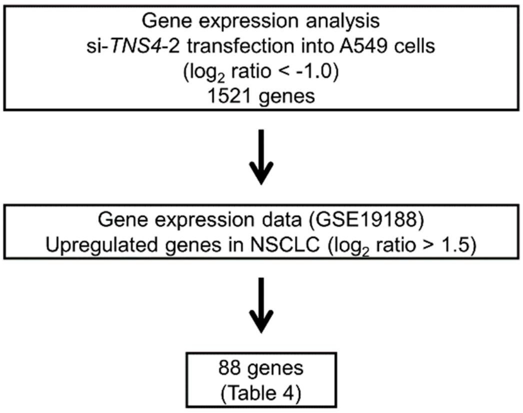

Figure S6. The strategy for identification of *TNS4*-modulated genes. We combined the gene expression analyses using A549 cells transfected with si-*TNS4* and GEO database (GSE19188). Finally, a total of 88 genes were identified as *TNS4*-modulated genes.

Table S1. Reagents used in this study.

**Antibody**	**Dilution**	**Catalog Number**	**Company**
TNS4	IHC 1:50<br>WB 1:500	ab82178	Abcam, Cambridge, UK
GAPDH	WB 1:20000	MAB374	EMD Millipore, Billerica, MA, USA
miRNA species	Concentration	Assay ID	Company
miR-150-5p	10 nM	PM 10070	Applied Biosystems, Foster City, CA, USA
miR-150-3p	10 nM	PM 12324	Applied Biosystems, Foster City, CA, USA
negative control miRNA	10 nM	AM 17111	Applied Biosystems, Foster City, CA, USA
anti-miR Negative Control #1	10 nM	AM 17010	Applied Biosystems, Foster City, CA, USA
siRNA	Concentration	Catalog number	Company
si-*TNS4*	10 nM	HSS131536<br>HSS131537	Invitrogen, Carlsbad, CA, USA
Primer and probe		Assay ID	Company
miR-26a		000405	Applied Biosystems, Foster City, CA, USA
miR-150-5p		000473	Applied Biosystems, Foster City, CA, USA
miR-150-3p		002637	Applied Biosystems, Foster City, CA, USA
RNU48		001006	Applied Biosystems, Foster City, CA, USA
TNS4		Hs00262662_m1	Applied Biosystems, Foster City, CA, USA
GUSB		Hs00939627_m1	Applied Biosystems, Foster City, CA, USA
Plasmid vector		Catalog number	Company

TNS4	RC222349	OriGene Technologies Inc., Rockville, MD, USA

Table S2. Immunohistochemical status and characteristics of the lung cancer and non-cancerous cases.

A. Immunohistochemical status and characteristics of LUAD cases

Patient no.	Grade	T	N	M	Pathological stage	Immunohistochemical intensity	Immunohistochemical extensity
21	1	1	0	0	IA	3	3
22	1	2	1	0	IIA	3	3
23	2	1	0	0	I	3	3
24	2	2	2	0	IIIA	2	3
25	2	3	1	0	IIIA	3	3
26	2	2	0	0	IB	3	3
27	2	3	2	0	IIIA	3	3
28	2	1	0	0	IA	2	3
29	2	3	1	0	IIIA	1	3
30	-	4	0	0	IIIA	3	3
31	2	2	0	0	IIA	1	3
32	2	3	1	0	IIIA	2	3
33	2	2	1	0	IIB	1	3
34	2	3	0	0	IIB	0	0
35	2	3	1	0	IIIA	2	3
36	3	2	1	0	IIB	3	3
37	3	2	0	0	IB	1	3
38	2	2	0	0	IB	1	3
39	-	2	0	0	IB	3	3
40	2	2	1	0	IIB	1	3

B. Immunohistochemical status of non-cancerous cases

patient no.	Immunohistochemical intensity	Immunohistochemical extensity
87	0	0
88	1	2
89	1	3
90	1	3
91	1	2
92	1	3
93	1	2
94	1	1
95	1	3
96	2	3
97	1	2

98	1	3
99	2	3
100	2	2

© 2019 by the authors. Submitted for possible open access publication under the terms and conditions of the Creative Commons Attribution (CC BY) license (http://creativecommons.org/licenses/by/4.0/).
